# The role of economic freedom and clean energy in environmental sustainability: implication for the G-20 economies

**DOI:** 10.1007/s11356-022-18666-5

**Published:** 2022-01-22

**Authors:** Andrew Adewale Alola, Uju Violet Alola, Saffet Akdag, Hakan Yildirim

**Affiliations:** 1 Department of Economics, School of Accounting and Finance, University of Vaasa, Vaasa, Finland; 2 Department of Economics and Finance, South Ural State University, Chelyabinsk, Russia; 3 Department of Tourism Guidance, Faculty of Economics, Administrative and Social Science, Istanbul Gelisim University, Istanbul, Turkey; 4 Department of Management, South Ural State University, Chelyabinsk, Russia; 5 Department of Banking and Finance, Faculty of Applied Sciences, Tarsus University, Tarsus, Turkey; 6 Department of Logistic Management, Istanbul Gelisim University, Istanbul, Turkey

**Keywords:** G-20, Clean energy, Environmental sustainability, Sustainable development, Economic freedom

## Abstract

With the increasing challenge of attaining sustainable balance in socioeconomic-ecosystem activities, the aspects of the global goals are continously being harnesed in order to ensure a sustainable interaction. As an alliance of the United Nations, the G-20 member countries have not only committed to attaining the Sustainable Development Goals 2030, the alliance body has further fostered frameworks that are targeted at advancing global economic and environmental sustainability. Within this context, the current study examined the environmental sustainability effects arising from the economic freedom prowess in the panel of the G-20 economies over the period 2000–2016. Among the sparse studies, the study employed the indices of economic freedom: freedom to trade internationally, regulation, sound money, legal framework, and property right and alongside the real income and renewable energy consumption as explanatory indicators. With the result of the difference- and two-step system GMM (generalized method of moments), the legal system and property right, sound money, freedom to international trade, and regulatory efficiency are detrimental to the panel countries’ environmental quality. Although this is likely to be untrue for countries that have advanced their climate actions and especially the Sustainable Development Goals (SDGs) 2030, it suggests a dearth in the SDGs achievement among the developing and emerging economies. Moreover, it probably shows the depth of traditional or business-as-usual practices (such as the lack of sustainable economic and environmental practices) and the socioeconomic system that are obtainable in most of the developing and emerging economies. Thus, the study put forward tangible policies that are essential for governance and toward attaining desirable country-specific SDGs.

## Introduction

The persistence drive toward a sustainable environment and development in spite of the dimensions of global challenges could only have yielded a relatively desirable outcome among the advanced economies. Although a few of the G-20 and G-7 member countries have consistently experienced economic growth in the last decade, the serious threat to environmental quality arising from the global warming has remained one of the greatest challenge to the sustainable development. Similarly, the increasing human exploitation of natural resources is mounting persistent pessure on the natural eclogical environment. These actions have resulted to a number of ecological complications such as environmental degradation, ecological degradation, and climate warming that are standing as threats to the economic growth and development globally (Alola et al. 2019c; Alola and Joshua 2020; Ulucak and Khan 2020; Wang et al. 2020; Adedoyin and Zakari 2020; Adedoyin et al. 2021). For instance, the report of World Energy Outlook (WEO) in 2017 linked the premature deaths of about 3 million to energy pollution especially from non-renewable energy sources. In specific, pollutant emissions from the use of fossil fuels, coal, and tradition cooking fuel from firewood among are increasingly constituting the highest source of emissions, thus causing serious environment to degradation.

More importantly, the environmental Kuznets curve (EKC) hypothesis depicts the (tarde-off and/or U-shaped) relationship between economic growth and environmental degradation in such a way that an increase in the later is triggered by an increase in the former at the early stage of development until a thresshold level is attained when the association between the two variables becomes negative (Grossman and Krueger 1991; Stern 2004). Following this perspective, the U-shaped relatiosnhip between economic output and environmental sustainabiity has been further conceptualized by augmenting the relationship with other social and economic indicators such as energy, trade, tourism, technology, health, and agriculture (Katircioğlu 2014; Shahbaz et al. 2014; Apergis and Ozturk 2015; Al-mulali et al. 2015; Ozturk et al. 2016; Higón et al. 2017; Asongu 2018; Sarkodie et al. 2020; Alola and Ozturk 2021).

However, the need to achieve a more rapid sustainable development should align with the enhancement of environmental quality and the ecosystem capabilities which plays a key role in human activities. In addition, the drive toward achieving the rapid economic growth amidst sustainable environment has further deepened the global need to preserve the world ecological footprint (EF), thus reducing the humans’ endangering activities. The ecological footprint as accounted by the framework of the Global Footprint Network (GFN) is measured as the demand for nature by humans and which amount to the number of natural resources humans use to support and meet their needs. The EF takes into consideration the comparison of the quantity of biologically produced goods that are available for human consumption and the biologically productive area of the geographical location under consideration. Considering that increasing number of countries is experiencing a deficit EF, this suggest the desirability of countries to shift toward the consumption of biodegradable or re-usable resources in the value chain of economic activity rather than non-renewable resources such as the fossil oil and other carbon-laden fuels.

For instance, among the G-20-member countries (Argentina, Australia, Brazil, Canada, China, France, Germany, India, Indonesia, Italy, Japan, Mexico, Republic of Korea (South Korea), Russia, Saudi Arabia, South Africa, Turkey, the UK, the USA, and the European Union (EU)), only 5 countries and the EU are currently not showing deficit in the EF (Global Footprint Network 2020). Specifically, the degree (in percentage) of how much the EF is greater than biocapacity is obviously severe in 14 of the G-20 economies: China (278%), France (87%), Germany (199%), India (173%), Indonesia (32%), Italy (371%), Japan (672%), Mexico (122%), Republic of Korea (797%), Saudi Arabia (1480%), South Africa (229%), Turkey (133%), the UK (301%), and the USA (122%). Interestingly, the above evidence is associated with the fact that the G-20 economies account for about 90% of the global GDP with the economic organization’s population amounting to about 60% of the world population. Considering the aforementioned motivation and that the G-20 economies contribute about 75% to the global greenhouse gases (GHGs) emissions (Climate Transparency 2016), the economic organization has further encouraged the members’ commitment to climate actions such as to put their carbon footprint under control, thus mitigating climate change and global warming globally.

In view of the above motivation, and as an objective, the current study is designed to examine the role of economic freedom on environmental sustainability amidst the drive for economic prosperity of the G-20-member countries. In order to achieve the desired objective, the study implements clean energy-driven growth (growth induced by renewable energy consumption) in addition to the economic freedom indicators that include legal systems and property rights, sound money, freedom to international trade, and regulation. By employing the ecological footprint as a proxy for environmental sustainability in a unique framework, the generalized method of moments (GMM) is employed for the panel of G-20 economies in a novel approach. Moreover, this study offers a significant contribution to the literature by employing a set of sparsely factors such as law and order and propoerty right, sound money, freedom of international trade, and regulations. Therefore, by exploring the sustainable development and environmental quality drive of the G-20 countries from the perspective of economic freedom, the contributions of the current study to the exising body of knowledge are considered a novel point of observation.

The subsequent sections are arranged in a specified order. The “Methodology,” “Findings and discussion,” and “Conclusion and policy suggestion” sections present the data and methodlogy, the discussion of the findings, and conclusion of the study, respectively.

## Methodology

### Description of indicators

This study employed the dataset described (see Table 1) that span over the period 2000 to 2016 for the balanced panel of the G-20 economies excluding the EU and Suadi Arabia.

**Table 1:** The description of the variables and statistics properties

Variable	EF	GDPc	RENE	LSPR	SMO	FT	REG
Measurement	Global hectare	Per capia	Thousands	0 to 10	0 to 10	0 to 10	0 to 10
	(Gha)	(Constant 2010 USD)	Tonnes	0 = low	0 = low	0 = low	0 = low
				10 = high	10 = high	10 = high	10 = high
Source	GFN	WDI	OECD	Fraser Institute1 Fraser Institute Fraser Institute Fraser Institute			
Statistics							
Mean	7.067	21,801.61	49,185.97	6.239	8.519	7.369	6.937
Minimum	4.493	443.314	758.031	3.009	3.567	3.600	4.120
Maximum	8.450	68,150.11	266,484.6	8.795	9.887	9.367	8.973
Standard deviation	0.799	17,802.44	63,580.17	1.453	1.381	0.930	1.137
Skewness	− 0.348	0.451	1.661	− 0.052	− 1.392	− 0.936	− 0.088
Kurtosis	2.523	1.866	4.568	1.802	4.663	5.309	2.225
Obserrvations	302	302	302	302	302	302	302

**Note**: The EF, GDPc, RENE, LSPR, SMO, FT, and REG are respectively the ecological footprint, gross domestic product per capita, renewable energy consumption, law and order and propoerty right, sound money, freedom of international trade, and regulations. Also, Organization for Economic Cooperation and Development is OECD, World Development Indicator (of World Bank) is WDI, and Global Footprint Network is GFN

^1^The economic freedom of the world index has consistently been reported by the Fraser Institute (2018)

### Model framework

Several decades ago, Ehrlich and Holdren (1971) and a similar follow-up by Holdren and Ehrlich (1974) both illustrated that the environmental impacts (I) of population growth (P), affluence (A), and technology change (T) could be expressed as IPAT (such that I = PAT). Consequently, York et al. (2003) later modified the IPAT into a stochastic model to imply that the environmental impact can be modeled as STIRPAT (for stochastic ımpacts by regression on population, affluence, and technology). However, that is not without an arguement that suggest that the importance of environmental impact of human behavior should not be undermined; thus, the study of Schulze (2002) rather advanced the IPAT model as IPBAT where B represents human behavior. Following the above representation of environmental impact, recent studies that augment the STITPAT model have been presented with the incorporation of relevant factors that potentially exhibit environmental impact. In the extant studies, the justification for economic advancement, technological innovation, population, and several aspects of human activities and evironmental-related nexus have been widely covered (Ardito et al. 2019; Cop et al. 2020, 2021; Aldieri et al. 2021).

Following the perspective from the provious studies that ecological footprint appropriately reflects and captures environmental impact relative to other environmental variables such as the greenhouse gas and carbon emissions emissions, the STIRPAT model is augmented by incorporating REG and LSPR as proxy for behavioral factors accordingly.

1 $$\mathrm{EF}=f(\mathrm{EF},\mathrm{ GDPc},\mathrm{ RENE},\mathrm{ LSPR},\mathrm{ SMO},\mathrm{ FT},\mathrm{ REG})$$

Primarily, the variables EF, GDPc, and RENE are transformed to natural logarithm while the other variables are employed directly since they are indexes. In additon, other priori tests implied that the variables are integrated after first difference (the result is not supplied for lack of space) and that there is significant evidence of cointegration (see Table 3 in Appendix).

#### Empirical method

This study is designed to examine the environmental effects of some selected indicators of economic freedom on the ecological footprint of the panle of G-20 economies. In this study, we settled for the difference GMM and system GMM. The technique is considered approapriate and the best estimation techniques for the study most importantly because the number of cross-section (number of examined countries in the panel), *N* = 18, is more than the number of period *T* = 17). Additionally, this approach is considered as to mitigate the effects of heteroscedasticity and endogeneity of the independent variables in the employed panel dataset. Moreover, the approach makes provisions for lagged endogenous variable as an explanatory variable to avoid the the possibility of endogeneity and also give a robust estimate of a large data set (see Usman et al 2019). However, in this case, we choose the system two-step of the two models ahead of one-step based on suitability because of the indicated diagnostic inference from the autocorrelation and the Sargan tests. The two-step GMM is written as follows:

2 $${{\mathrm{ln}EF}_{it}= {a}_{0 }+{a}_{1 }\mathrm{ln}{EF}_{it} + {a}_{2}\mathrm{ln}{GDPc}_{it}+ {a}_{3}\mathrm{ln}{RENE}_{it}+ {a}_{4}{LSPR}_{it}+{a}_{5}{SMO}_{it}+ {a}_{6}FT}_{it}+ {a}_{7}{REG}_{it}+ {\varepsilon }_{it}$$

There are two ways of estimating GMM, difference GMM, and system GMM. For the difference GMM, Eq. (1) can be rewritten as follows:

3 $$\mathrm{ln}{EF}_{it}- {\mathrm{ln}EF}_{i,t-1}= {a}_{1 }\left({\mathrm{ln}EF}_{t-1}- {\mathrm{ln}EF}_{i,t-2}\right)+{a}_{2}\left(\mathrm{ln}{GDP}_{it}-\mathrm{ln}{GDP}_{i,t-1}\right)+ {a}_{3}(\mathrm{ln}{RENE}_{it}- \mathrm{ln}{RENE}_{i,t-1})+ {a}_{4}{(LSPR}_{it}- {LSPR}_{i,t-1})+ {a}_{5}{(SMO}_{it}- {SMO}_{i,t-1})+ {a}_{6}{(FT}_{it}- {FT}_{i,t-1})+ {a}_{7}{(REG}_{it}- {REG}_{i,t-1})$$

The condition for the difference GMM estimator in Eq. (3) is given below:

4 $$E\left({\mathrm{ln}EF}_{i,t-z}* {\varepsilon }_{i,t}\right)=0, for z\ge 2001 t=2000, 2001\dots \dots \dots .. 2016$$

Difference GMM is an effective estimator to control some distinct features of each of the countries and the endogenous independent variables. However, it could lead result to a biased parameter estimation in small sample space and larger variance asymptotically (Khan et al 2019). To circumvent the problems, another approach is system GMM. The condition for the system GMM for Eq. (2) is given below:

5 $$E\left({\mathrm{ln}EF}_{i,t-z}+ {\varepsilon }_{i,t-z-1}* {\varepsilon }_{i,t}\right)=0, for z=1, t=2002\dots ..\dots 2016$$

From Eq. (2) above, EF is the ecological footprint which is the dependent variable in the equation. GDP, RENE, LSPR, SMO, FT, and REG are the explanatory variables representing economic growth, renewable energy consumption, legal systems and property right, sound money, freedom of trade, and regulation, respectively. 〖EF〗_(i,t-1) is the first lag of the dependent variable and used as an estimate for measurement of the current year using previous years and ε_it is the stochastic disturbance that put into consideration uncaptured variables among the independent variables. The result of both the difference and system GMM is implied in Table 2.

**Table 2:** The result of the difference and system GMM

Explanatory variables	Difference GMM		System GMM	
	One-step result	Two-step result	One-step result	Two-step result
EF_−1_	0.199 (0.000)	0.147** **(0.050)**	0.268 (0.000)	0.278* **(0.000)**
lnGDPc	0.0369 (0.040)	0.0377 (0.158)	0.019 (0.082)	0.0651 (0.104)
LnRENE	− 0.014 (0.580)	0.013 (0.806)	0.058 (0.000)	0.088** **(0.038)**
Legal systems & property rights	0.226 (0.000)	0.278* **(0.000)**	0.123 (0.000)	0.189* **(0.000)**
Sound money	0.150 (0.000)	0.159* **(0.000)**	0.140 (0.000)	0.094* **(0.034)**
Freedom to trade	0.262 (0.000)	0.255* **(0.000)**	0.220 (0.000)	0.259* **(0.000)**
Regulation	0.206 (0.000)	0.179* **(0.000)**	0.126 (0.000)	0.137* **(0.000)**
Wald test	3653.04 (0.000)	1518.85* (0.000)	311,832.91 (0.000)	443.47* (0.000)
Sargan test	240.146 (0.000)	12.713 (0.997)	373.548 (0.000)	10.700 (0.998)
Ar _(1)_ probability Ar _(2)_ probability	0.000 0.951	0.007 0.893	0.004 0.838	0.037 0.821

***, **, and * indicate 1%, 5%, and 10% levels of significance, respectively

## Findings and discussion

According to the results obtained and indicated in Table 2, the consistency of GMM estimators is evaluated. The Wald test results implied that one-step models of both difference GMM and system GMM estimators are meaningful as a whole while Sargan test is not valid for over identifying restrictions in related models. Therefore, illustrated models (one-step models of both difference GMM and system GMM) are excluded or ignored. In addition to the two-stage test results, Wald test results show that of both two-step of the difference GMM and system GMM estimators are meaningful as a whole. However, the Sargan test implied that the over identifying restrictions in the system, two-step model is found to be valid. The autocorrelation test also provided additional evidence that shows that there is no autocorrelation between the variables in the AR (2) process. Foremost, the result of both the two-step (difference and system) GMM implied that the (first) lag of the ecological footprint positively affects the ecological footprint of the panel countries.

Additionally, the results of both the difference GMM and system GMM highlight that GDP per capital affects the ecological footprint positively. However, the impact is not significant; it specifically shows that the difference GMM posited a rise in GDP per capita by 1% and increases the ecological footprint by 0.038% while the system GMM indicates that an increase in GDP per capita by 1% raises the value of the ecological footprint insignificantly by 0.65%. This implies that increasing the individual income level of the people in the G-20 economies will worsen the environmental degradation because it will cause more demand on the country’s ecological footprint in G-20 countries. This result partially concurs with the fact that an improved income level of the individual might not necessarily trigger the consumption of goods and services that are detrimental to the environment. However, the result contradict the findings of Alola et al. (2019a, b, c) that found that a rise in the real income in the three largest European states worsens the environmental quality of the countries (France, Germany, and the UK).

Concerning renewable energy consumption, the results obtained from both the difference and systems GMM verify a positive impact of renewable energy consumption on the ecological footprint of the panel of G-20 economies. However, the impact is only significant at 10% level of significance for the system GMM model. More precisely, a 1% rise in renewable energy consumption increases the ecological footprint insignificantly by 0.13% for the difference GMM while the system GMM model shows that a 1% increase in the renewable energy consumption increases the ecological footprint of G-20 economies significantly by 0.88%. This implies that renewable energy utilization is yet to improve the environmental quality of the G-20 economies. Similar to the recent findings of Alola and Joshua (2020), the reason for this undesirable result could be associated with the fact that the components of renewable energy in most of the examined countries might not be totally from clean energy sources. Alola and Joshua (2020) found a positive relationship between CO_2_ emissions and renewable energy consumption, first for the lower middle-income economies and secondly for the panel of upper middle, lower middle, low-income, and high-income economies. Moreover, the renewable energy sources are mostly from geothermal, solid biofuels, biogasoline, biodiesels, other liquid biofuels, biogases, and others (Organization for Economic Cooperation and Development, OECD 2020). The results obtained in this regard contradict the findings expressed in the case of the EU countries (Alola et al. 2019b; Bekun et al. 2019; Adedoyin et al. 2020) that posited that renewable energy consumption improves the environmental quality in 16 EU economies. Moreover, there are other related studies that have also supported the negative nexus of renewable energy consumption and environmental sustainability (Bhattacharya et al. 2016; Bekun et al. 2020; Sharif et al. 2020).

Furthermore, the results also confirm that legal systems and property rights have a significant and positive impact on the ecological footprint of the G-20 economies. Specifically, the difference and system GM show that an increase in the legal systems and property rights increases the ecological footprint of G-20 economies significantly. This implies that improving the effectiveness of the judicial, increasing the accountability and independence of the G-20’s legal systems in addition to improving the property rights poses threat to the environmental quality of examined economies. The economic intuition can be viewed from the perspective that improved legal system and property right would amount to increased investment and economic opportunities thus accumulating more demand on the ecosystem. It is note mentioning that increased ecological stress will unavoidably prompt more environmental damage. Similarly, we also found a positive and significant impact of sound money on EF across the two-step model employed in the study. This implies that encouragement of sound money (a more stable currency) that neither easily appreciates nor depreciates due to business cycle or economic fluctuations deepens the threats on environmental sustainability of the panel of G-20 economies. Although the result is not desirable, there is high tendency that a more stable currency will be a huge interest to investors, in that more economic activities are triggered in such situation. However, the finding is in contrary to the evidence that was posited by Hashmi and Alam (2019).

Moreover, the results highlight that (freedom to international trade/open market) allowing free trade has a significant and positive impact on the ecological footprint in G-20 economies. The same observation is revealed for the impact of regulations (this constitutes business, labor, and monetary freedom) on the ecological footprint. This implies that allowing a free international trade and regulatory efficiency in the panel countries worsens the environmental quality. In both circumstances (when international trade is unhindered and high regulatory efficiency is encouraged), environmental degradation will worsen in G-20 economies especially because trade in environmentally hazardous goods and service is either unhindered or with limited restriction across borders. Except in the case of advanced economies that have consistently incorporated carbo actions as a policy across the economic sectors, the result is an indication that major economic activities of many of the member countries are largely driven by the business-as-usual approaches such as the use of conventional energy. These findings especially that hints on the environmental effect associated with regulatory quality, trade openness, and related policy are consistent with the findings of Ahmed and Ozturk (2019, Alola (2019), and Adedoyin et al. (2020).

## Conclusion and policy suggestion

For the first time in the literature, this study explored the economic freedom attributes associated with the G-20 economies from the perspectives of sustainable development vis-à-vis sustainable income and environmental sustainability. In this case, the role of the attributes of legal system and property right, sound money, freedom to international trade, and regulatory efficiency in environmental sustainability over the period of 2000 to 2016 is examined with the generalized method of moments. In order to control for other factors, the real income per capita and renewable energy consumption were utilized alongside the aforementioned economic freedom indicators. Interestingly, the impact of the aforementioned indicators (legal system and property right, sound money, freedom to international trade, and regulatory efficiency) on ecological footprint were all found to be significant and positive in both the two-step difference and system GMM estimations. Considering that these results are not environmentally desirable, relevant policy is essential in the panel countries to enhance a feasible attainment of the country-specific sustainable environment and development as outlined in the SDGs for 2030.

As a policy, the G-20 countries especially the developing and emerging economies should further incorporate sustainable economic and environmental policies across the sectors of their respective economies. The impact of legal system and property right, sound money, freedom to international trade, and regulatory efficiency on environmental sustainability could be better fashioned with frameworks that are targeted to have a more sustainable outcome. For instance, free international trade/open market and property right policies such as tax exemption or subsidy that encourages new investors and business owners could be adopted by any of the examined countries that are obviously slow toward carbon action commitments. In addition, the implementation of more stringent environmental policy such as the emissions trading system (ETS) and carbon tax across could offer more efficient approach in preventing threat of carbon-outsourcing or carbon leakages. In regard to sound money or the stabilization of the countries exchange (monetary) policy, re-engineering the countries’ major sectors on the framework of green economy is expected to provide a more balanced and desirable economy-environmental sustainability mechanism.

## Appendix

**Table 3:** Evidence of cointegration by Pedroni residual and Kao test

Pedroni residual cointegration Alternative hypothesis: common AR coefs. (within-dimension)					
		Statistic	Prob	Weighted statistic	Prob
Panel v-statistic		− 1.481	0.930	− 2.068	0.981
Panel rho-statistic		4.215	1.000	4.142	1.000
Panel PP-statistic		− 2.860	0.002^a^	− 3.322	0.000^a^
Panel ADF-statistic		− 3.844	0.000^a^	− 4.274	0.000^a^
Alternative hypothesis: individual AR coefs. (between-dimension)					
		Statistic	Prob		
Group rho-statistic		5.740	1.000		
Group PP-statistic		− 4.510	0.000^a^		
Group ADF-statistic		− 4.382	0.000^a^		
Kao residual cointegration test					
			t-Statistic	Prob	
ADF			− 5.824130	0.000^a^	
Residual variance			8.09E-05		
HAC variance			6.37E-05		

represents the 1% statistically significant level
